# Pre-, pro-, syn-, and Postbiotics in Infant Formulas: What Are the Immune Benefits for Infants?

**DOI:** 10.3390/nu15051231

**Published:** 2023-02-28

**Authors:** Anaïs Lemoine, Patrick Tounian, Karine Adel-Patient, Muriel Thomas

**Affiliations:** 1 Pediatric Nutrition and Gastroenterology, Trousseau Hospital, Assistance Publique—Hôpitaux de Paris, Sorbonne Université, F-75012 Paris, France; 2 UMR1319, Micalis Institute, INRAE, AgroParisTech, Université Paris-Saclay, F-78350 Jouy-en-Josas, France; 3 Paris Center for Microbiome Medicine (PaCeMM) FHU, AP-HP, F-75571 Paris, France; 4 Département Médicaments et Technologies pour la Santé (DMTS), SPI/Laboratoire d’Immuno-Allergie Alimentaire, Université Paris-Saclay, CEA, INRAe, F-91190 Gif-sur-Yvette, France

**Keywords:** prebiotic, probiotic, synbiotic, postbiotic, infant formula, immunity, atopy, microbiota

## Abstract

The first objective of infant formulas is to ensure the healthy growth of neonates and infants, as the sole complete food source during the first months of life when a child cannot be breastfed. Beyond this nutritional aspect, infant nutrition companies also try to mimic breast milk in its unique immuno-modulating properties. Numerous studies have demonstrated that the intestinal microbiota under the influence of diet shapes the maturation of the immune system and influences the risk of atopic diseases in infants. A new challenge for dairy industries is, therefore, to develop infant formulas inducing the maturation of immunity and the microbiota that can be observed in breastfed delivered vaginally, representing reference infants. *Streptococcus thermophilus*, *Lactobacillus reuteri* DSM 17938, *Bifidobacterium breve* (BC50), *Bifidobacterium lactis* Bb12, *Lactobacillus fermentum* (CECT5716), and *Lactobacillus rhamnosus* GG (LGG) are some of the probiotics added to infant formula, according to a literature review of the past 10 years. The most frequently used prebiotics in published clinical trials are fructo-oligosaccharides (FOSs), galacto-oligosaccharides (GOSs), and human milk oligosaccharides (HMOs). This review sums up the expected benefits and effects for infants of pre-, pro-, syn-, and postbiotics added to infant formula regarding the microbiota, immunity, and allergies.

## 1. Introduction

A host and its commensal microbiota live in symbiosis, allowing both the establishment of local immunity and maturation of the intestinal epithelium [1,2]. The development of the intestinal microbiota at birth is progressive and sequential. The microbiota matures during the first years of life until reaching a kind of “status-quo” after 3 years. The main characteristic of the primo-colonizing pattern at birth is high inter-individual variability, reflecting the fragile acquisition of a diverse ecosystem. Colonization becomes massive after birth [3]. It starts with *Enterobacteriaceae*, then *Bifidobacterium*, *Bacteroides*, and *Clostridium* [3,4].

Depending on the type of birth, the early microbiota of infants differs, with a gut microbiota close to the mother’s vaginal microbiota in the case of vaginal delivery and one close to the mother’s skin microbiota for cesarean births [3,5]. Infants born via C-section have more *Clostridium* and pathogenic potential bacteria and less *Bifidobacteria* and *Bacteroides* [4]. Significant variations in the microbiota due to the type of birth disappear between 6 and 14 months [6].

Breastfeeding remains the strongest factor influencing the digestive microbiota of infants in the first year of life [6]. *Bifidobacteria* usually represent the dominant taxon (up to 90%) in breastfed infants delivered vaginally [4]. Breastfeeding provides *Bifidobacterium* sp., *Lactobacillus* sp., and *Staphylococcus* sp. naturally present in mothers’ milk. More importantly, breastfeeding promotes the implantation of *Bifidobacteria* thanks to the richness and high diversity of human milk oligosaccharides (HMOs), which are uniquely metabolized by the bacteria. In a virtuous circle, endogenous synthesis of secretory IgA (sIgA) by the intestinal mucosal lymphocytes into the lumen is also conditioned by the presence of microbiota, particularly *Bifidobacteria* [7], after the first weeks of life when sIgA can only be provided by breastmilk. sIgA is an important weapon in immune defense against pathogens and toxins [2].

Conversely, formula-fed infants have a faster maturation of their gut microbiota compared to breastfed infants. Indeed, microbiota from formula-fed infants is diversified earlier, resulting in an enrichment in anaerobic bacteria, such as *Bacteroides* and *Clostridium*, with a lower representation of so-called “beneficial” bacteria, such as *Bifidobacteria* and *Lactobacilli* [8].

Overall, a lower abundance of *Bifidobacteria,* as observed in cesarean-born or formula-fed infants, is a risk factor for impaired metabolism of short-chain fatty acids (SCFAs), an increase in stool pH, and a weakening of the intestinal barrier function. As a result, the dialogue between the microbiota and the host is disturbed, the risk of colonization by pathogens is greater, and digestive inflammation can be observed. All these parameters may also participate in altered immune system programming and metabolic disorders. These infants have an increased risk of developing immune-related disease, such as allergic diseases, autoimmune diseases, or other chronic digestive or extradigestive diseases [4,5].

For several reasons, some newborns and infants cannot benefit from breastfeeding. The objective of dairy industries is then to ensure that infant formulas are as close as possible to breastmilk, both in its composition and its physiological properties. Some breastmilk bioactive components are unique and specific to human milk, and some, such as cytokines and growth factors, are associated with health outcomes in infancy (e.g., food allergies [9]). However, their addition to infant formula is not planned to date (due to cost and stability). On the other hand, supplementation with prebiotics or health-promoting (live) bacteria seems a more rational and easier approach to improve the health-promoting capacity of formulas. Since breastfed infants have more *Bifidobacterium* in their microbiota, the first strategy was to add probiotics and, in particular, *Bifidobacteria* directly into infant formulas, followed by prebiotics and, more recently, synbiotics and postbiotics for their bifidogenic effects, as well as for their own positive expected effects on immunity. Nowadays, more than half of formula-fed infants consume probiotic-enriched formula in France [10]. The goal of this review is to sum up the pre-, pro-, syn-, and postbiotics (named “-biotics” in this review) used in infant formulas and the expected and proven clinical benefits for infants regarding microbiota composition, immunity, and allergies.

## 2. Methods

**Figure 1:**
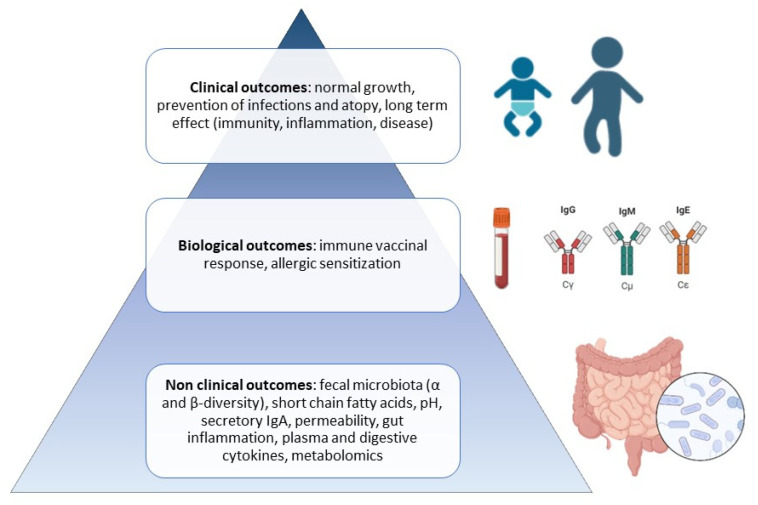
Pyramid of preclinical and clinical expected outcomes of pre-, pro-, syn-, and postbiotics during infancy.

To establish the current knowledge on “-biotics” in infant milk, a literature search was conducted until December 2022 using PubMed^®^ databases with a combination of keywords: “prebiotic”, “probiotic”, “synbiotic”, “postbiotic”, or “human milk oligosaccharides”, and “infant milk” or “formula”. The researched article types were “randomized controlled trial” and “clinical trial”. Articles mainly published over the last 10 years (since 2012) were evaluated based on their title and abstract, checking for the inclusion and exclusion criteria. We focused only on primary and secondary outcomes at a nonclinical level regarding the microbiota, digestive metabolites, and intestinal immunity; at a biological level, including serum immune biomarkers; and finally, clinical outcomes, such as infection, inflammation, atopy, and allergy in infancy (Figure 1). Some older articles were added to the current review if they were quoted in the newest articles. We did not retain in this review outcomes regarding growth, non-allergic, or non-infectious digestive symptoms, such as infant colic for example. We excluded clinical trials in preterm infants and studies on the supplementation of “-biotics” as a medication or added into a diet other than infant milk. We also excluded animal studies if the results were not transposable or proven in humans. The bacterial strain names were used as in the original publications.

## 3. Results

### 3.1. Probiotics

#### 3.1.1. Definition

Probiotics are live microorganisms with a recognized presumption of safety and, when they are administered in adequate amounts, they confer a health benefit to a host [11]. Some authors refer to newly described commensal bacteria as “next-generation probiotics”. These bacteria are usually isolated from the human gut or traditional fermented foods, have a long co-evolution with humans, and are generally associated with “good health”, i.e., present in controls, deficient in patients, and restored after treatment [12]. The EFSA (European Food Safety Authority) controls the use of the term “probiotic”, and this regulation authority considers the mention of probiotics in a food to imply a health claim demonstrated by clinical studies. Therefore, in Europe there is a gap between the huge number of products rich in ferments available on the market, the expectations of consumers, the innovation potential of companies, and the limitations of regulatory agencies. In the USA, the FDA (Food and Drug Administration) considers probiotics as nutritional supplements containing live microbials without any specific health claim and not as pharmaceuticals that need to be approved.

The QPS (Qualified Presumption of Safety) program in Europe and the GRAS (Generally Recognized as Safe) status in the USA provide safety assessments.

Probiotics in infant formula added in adequate amounts are safe and ensure normal growth in healthy infants during infancy [13].

#### 3.1.2. *Bifidobacterium animalis* sp. *lactis* Bb-12 and *B. lactis* CNCM I-3446

*Bb lactis* Bb-12 is a bacterium originally isolated in fermented milk that inhabits the guts of healthy adults and infants.

*B. lactis Bb-12* (10^6^ colony-forming units (CFUs)/g) given for 6 weeks to 6-week-old infants (*n* = 50) had immunomodulatory properties and stimulated the production of digestive sIgA [14]. The risk of acute gastroenteritis and its severity were lower in infants under 8 months of age who were cared for in community and received *B. lactis* Bb-12 through infant formula (*n* = 46) when compared to non-supplemented and non-breastfed infants (*n* = 44) [15]. After cessation of supplementation, *B. lactis* Bb-12 did not persist in infant stool, and fecal sIgA decreased [16].

Infants born via C-section and fed with an infant formula supplemented with *B. lactis* Bb-12 (low dose: 3.7 ± 2.1 × 10^4^ (*n* = 84); regular dose: 3.1 ± 1.4 × 10^7^ CFU/g powder (*n* = 80)) from 0 to 6 months had fecal *Bifidobacteria* levels similar to those found in breastfed and C-section-born infants. The prevalence of acute infectious gastroenteritis was similar in all the groups. The fecal biomarkers (calprotectin and alpha-1-antitrypsin) were also comparable. As expected, breastfed children had higher fecal sIgA during the first 4 months [17]. Nevertheless, in this efficacy pilot study, a control group of caesarean-born and formula-fed infants without probiotics was missed and prevented gaining more robust conclusions on infection prevention.

In a large, nationwide French observational cohort, consumption of *B. lactis* Bb-12-enriched formula between 2 and 10 months was associated with a reduced risk of lower respiratory tract infection (LRTI) and asthma up to 5.5 years of age [10].

#### 3.1.3. *Lactobacillus casei* CRL431 and *B. lactis* Bb-12

A combination of *Lactobacillus casei* CRL431 and *B. lactis* Bb-12 (10^7^ CFU/g for formula for each) added in an extensively hydrolyzed casein formula (eHCF) was tested in infants allergic to cow’s milk (eHCF + probiotic (*n* = 53) vs. eHCF alone (*n* = 57)). The probiotic supplementation failed to accelerate the acquisition of tolerance to cow’s milk proteins [18]. In a post hoc analysis, Dupont et al. showed that all the allergic subjects improved their SCORAD (Scoring Atopic Dermatitis) index, reflecting a decrease in the clinical activity of atopic dermatitis with this hydrolyzed formula, but no significant effect could be attributed to the probiotic supplementation [18].

#### 3.1.4. *Lactobacillus paracasei* sp. paracasei, strain F19 (F19)

In a study published in 2021, Li et al. showed an immunomodulatory capacity of F19. Indeed, 4-month-old healthy infants fed with F19 formula (10^8^ CFU/L) (*n* = 195) from the third week of life had greater serum levels of IL-2 and lower levels of IFN-γ compared to infants fed standard formula (*n* = 194), as well as higher serum concentrations of IL-2, IL-4, and IL-17A than breastfed infants (*n* = 208). However, vaccine responses were similar between the formula and breastfed groups, and clinical consequences such as infectious events were not described by authors [19].

#### 3.1.5. *Lactobacillus reuteri* DSM 17938

*L. reuteri* DSM 17938 has been isolated from mother’s milk and is associated with the prevention of colic in breastfed infants [20]. This strain is marketed both as a food supplement in the form of drops to be given orally daily, as well as directly added in certain infant formulas.

Newborns and infants fed an infant formula supplemented with this strain (1.2 × 10^9^ CFU/L) (*n* = 20) had a higher relative proportion of *Lactobacillus* in fecal extracts collected after 2 weeks and 4 months of the intervention when compared to the non-supplemented group (*n* = 20). Notably, at 2 weeks, there was an increase in the *Bifidobacterium* genus in stool from supplemented newborns delivered via C-section (*n* = 10) similar to the microbial composition of non-breastfed infants (supplemented or not) delivered vaginally (*n* = 20). Conversely, non-supplemented infants delivered by C-section (*n* = 10) evidenced less fecal *Bifidobacteria* and more *Enterobacteria.* The underlying hypothesis was that supplementation with *Lactobacillus reuteri* DSM 17938 at an early stage of microbiota maturation could acidify the intestinal lumen through lactate production. Such acidification favored the growth of *Bifidobacteria* to the detriment of *Enterobacteria,* which are acid-sensitive. This, thus, allowed a more rapid attenuation of the dysbiosis induced by caesarean birth [21].

No association was made between consumption of this strain in infant formula and risk of respiratory diseases up to 5 years old [10].

#### 3.1.6. *Lactobacillus rhamnosus* GG (LGG)

In cow’s-milk-protein-allergic infants (*n* = 55 to 365), supplementation of an eHCF with LGG (at least 1.4 × 10^7^ CFU/100 mL) had positive effects on digestive inflammation, accelerated the acquisition of tolerance to cow’s milk, improved functional bowel disorders, and limited the “atopic march” in infants at 12, 24, and 36 months [22,23,24,25,26]. This eHCF and LGG combination positively influenced microbiota function, with an increased production of butyrate, known to modulate the acquisition of immune tolerance [27,28]. The underlying mechanisms were partly due to epigenetic modifications of genes involved in immune regulation favoring protolerogenic pathways: demethylation of the FOXP3 gene in regulatory T cells (Treg), increased expressions of IL-10 and IFN-γ cytokines, and decreased expressions of pro-allergenic IL-4 and IL-5 cytokines [29,30]. Even if the effects on food allergy remission seemed encouraging, after a 5-year follow up, no difference was observed in the incidence of infections among infants receiving eHCF and LGG (*n* = 32), partially hydrolyzed formula and LGG (*n* = 36), and eHCF without LGG (*n* = 28) [31].

The effects of a partially hydrolyzed cow’s milk protein infant formula with and without LGG (10^6^ CFU/g) on stool microbiome and gut inflammation were evaluated in neonates (inclusion between 14 and 28 days of age) with infantile colic (*n* = 35 in the LGG group; *n* = 36 in the control group). The intervention period lasted 3 weeks. As expected, the relative abundance of LGG was higher in the LGG group vs. the control group and vs. baseline. At the end of the study, the alpha diversity (measure of the microbiome diversity applicable to a single sample) was lower than that of control group. Fecal calprotectin was not different between the groups and over time [32].

#### 3.1.7. *Lactobacillus fermentum* CECT5716

*L. fermentum* CECT5716 was first isolated from four-day-postpartum human milk and then characterized as a probiotic in humans [33].

In a randomized cohort of healthy infants aged from 1 to 12 months, the incidence and duration of diarrhea were 44% lower (*p* = 0.014) and 2.5 days shorter (*p* = 0.044), respectively, in a group of infants supplemented with 10^7^ CFU/g of *L. fermentum* CECT5716 Lc40 (*n* = 65) compared to infants receiving non-supplemented formula (*n* = 61) [34]. A higher load of *Bifidobacterium* in feces was related to a lower risk of diarrhea (OR = 0.76, *p* = 0.027) [34].

Conversely, in an ELFE cohort, discontinued consumption of *L. fermentum* between 2 and 10 months of life was associated with a higher risk of upper respiratory tract infection (OR 1.21; 95% CI [1.02–1.44]), whereas daily consumption did not confer any additional risk [10].

#### 3.1.8. *Bifidobacterium breve* CECT7263

As with previous strains, *B. breve* CECT7263 was isolated from human milk.

No differences in the incidence and duration of respiratory and gastrointestinal infections were observed between infants supplemented with 10^7^ CFU/g of *B. breve* CECT7263 during the first year of life (*n* = 63) and infants receiving the control formula (*n* = 61) [34].

#### 3.1.9. *Bifidobacterium longum* sp. *infantis* CECT7210 (*B. infantis* IM1)

The effect of *B. infantis* IM1 supplementation (10^7^ CFU/g) was explored in healthy infants recruited before 3 months of age and receiving formula for 12 weeks (*n* = 93 in probiotic group; *n* = 97 in control group) [35]. A non-significant decrease in diarrhea events per infant was observed in the supplemented group (probiotic group: 0.05 ± 0.28 vs. control: 0.29 ± 1.07, *p* = 0.059). Even if fecal sIgA concentrations were similar in both groups, a linear regression model revealed that *B. infantis* IM1 could modulate sIgA concentrations at the end of the intervention [35].

#### 3.1.10. *Bifidobacterium animalis* sp. *lactis* HN019

Dekker et al. randomized healthy infants aged from 6 to 12 months into three groups (*Bifidobacterium animalis* sp. *lactis* HN019, 10^6^ CFU/g (*n* = 64); *Lacticaseibacillus rhamnosus* HN001, 10^6^ CFU/g (*n* = 64); control group: same infant formula without added probiotics (*n* = 64)) to compare bacterial and viral infections during winter. Over a 12-week period, in comparison with the control group, infants consuming HN019 had fewer physician-confirmed infections (*p* = 0.029), fewer parentally reported infections (*p* = 0.019), and lower use of antibiotics (not significant) [36].

#### 3.1.11. *Lacticaseibacillus rhamnosus* HN001

According to the same study [36], *Lacticaseibacillus rhamnosus* HN001 failed to decrease winter infections, with similar rates of physician-confirmed infections and parentally reported infections as those found in the control group (*p* = 0.3 and *p* = 0.1, respectively) [36].

#### 3.1.12. Other *Bifidobacteria*

During a 1-year study started from birth, Bazanella et al. showed that fecal metabolites and microbiota data discriminated stool from infants fed an intervention formula supplemented with a mix of *Bifidobacteria* (10^7^ CFU/g of *B. bifidum*, *B. breve*, *B. longum*, and *B. longum* sp. *infantis*) (*n* = 48) and that from non-supplemented infants (*n* = 49). The relative abundance of fecal *Bacteroides* *fragilis* and *Blautia spp*. decreased in the intervention group, which was associated mainly with changes in lipid metabolites. Even if fecal metabolites were clearly distinct during the first months between infants receiving the intervention formula and breastfed infants, their profiles converged over time. Any strains of the intervention formula colonized the infant gut at month 24, 1 year after the end of supplementation. No significant differences were observed between the infant feeding groups regarding infantile disease (fever, diarrhea, and antibiotics) [37].

These results suggest that, even if the microbiota composition and function could be modulated in early life with a *Bifidobacteria*-supplemented formula, no detectable mid-term consequences were observed.

Table 1 summarizes the main clinical effects of probiotics in infant formula.

### 3.2. Prebiotics

#### 3.2.1. Definition

Prebiotics are indigestible substrates for humans but are metabolized by host microorganisms and exert a beneficial effect on health [38,39]. They can selectively stimulate the growth or activity of specific bacteria and, thus, promote the production of SCFAs, which have pleiotropic effects both locally, i.e., in the intestinal tract, and at distance on other tissues [40,41]. European regulations do not allow the mention of prebiotics on food packaging and the related health claim without an established and proven effect by clinical studies. In the USA, prebiotics have no legal definition from the FDA (Food and Drug Administration).

Prebiotics are naturally present in many fiber-rich foods. The most common prebiotics are carbohydrate-based, such as resistant starch, cellulose, pectin, and fructan, as well as oligosaccharides structured in fructo-oligosaccharides (FOSs) and galacto-oligosaccharides (GOSs). Breastmilk also contains a large number of natural prebiotics, i.e., human milk oligosaccharides (HMOs). Dietary fibers have numerous demonstrated direct and indirect health benefits through the fiber–microbiota–immune relationship. The main bacterial metabolites coming from the fermentation of fibers are SCFAs (mostly acetate, butyrate, and propionate), which are potent immunomodulators associated notably with allergy protection [42]. Prebiotics added in adequate levels to infant formula are well-tolerated and ensure normal growth [43]. Adverse events can be observed at high levels of consumption.

#### 3.2.2. HMOs

HMOs are the third most prevalent component of human milk, after lactose and lipids [44]. They are indigestible carbohydrates that selectively stimulate the colonic growth of HMO-consuming bacteria, including *Bifidobacteria* [45,46]. More than 200 different HMOs have been identified in human milk, with up to 130 for an individual mother. HMO composition is highly influenced by the genetic status of the mother, i.e., secretor and Lewis statuses determining the expressions of FUT2 and FUT3 fucosyltransferases, respectively. As a result of FUT2 activity, 2’-fucosyllactose (2’FL) is the most abundant HMO in breastmilk from secretor mothers (70–90% depending on country), representing 20–40% of the total HMO concentration in colostrum [45]. HMOs promote intestinal barrier function, prevent adhesion of pathogens to epithelial cells, act as decoy receptors, and stimulate the development of an infant’s immune system either directly or through a microbiota-mediated effect [47]. Globally, HMOs may then help in preventing infections and diseases related to immune dysregulation, such as allergic and autoimmune diseases [45,47]. It is still unclear whether the protective effect of HMOs is specific to certain classes of HMOs [45] or if it relies on their high diversity and synergic actions. To date, due to technical difficulties and cost issues, only a few HMOs have been synthetized, i.e., 2’FL, 3-fucosyllactose (3FL), 3′-sialyllactose (3′SL), 6′-sialyllactose (6′SL), and lacto-N-neotetraose (LNnT), for use as supplements in infant formulas.

In vitro studies evidenced that 2’FL increased the relative proportions of *Bifidobacterium adolescentis* and other bacteria that produce butyrate, a beneficial SCFA [47]. 2′FL also reduced the adhesion of pathogens such as *Clostridium difficile*, *Campylobacter jejuni*, enteropathogenic E. coli, and *Pseudomonas aeruginosa* to epithelial cells [47]. In infants, supplementation with 2′FL promoted the growth of *Bifidobacterium* species and limited the colonization of opportunistic pathogens, such as *C. difficile* and *K. pneumonia* [46].

Feeding with a formula supplemented with 2’FL and GOS (2.4 g total oligosaccharides/L: 2′FL at 0.2 g/L with GOS at 2.2 g/L (*n* = 54) or 2′FL at 1 g/L with GOS at 1.4 g/L (*n* = 48)) for 6 weeks resulted in inflammatory cytokine profiles in the plasma that were intermediate between that of infants fed with control infant formula (GOS only, 2.4 g/L, *n* = 48) and that of exclusively breastfed infants (*n* = 51) [48].

In healthy infants, the use of infant formulas enriched with 2’FL (1 g/L) and LNnT (0.5 g/L) (*n* = 88, vs. *n* = 87 in the control group) during the first 6 months of life was associated with a decrease in lower respiratory infections and with the use of antibiotics and antipyretics before the age of 1 year, but these results were the secondary endpoints of a tolerance study [49]. At 3 months, fecal microbiota compositions (alpha diversity; beta diversity; relative abundance of *Bifidobacterium*, *Escherichia*, unclassified *Peptostreptococcaceae,* and *Streptococcus*) of infants supplemented with HMOs were closer to that of breastfed children than that of the control group. HMOs increased the proportion of infants with a fecal community type characterized by high abundance of *Bifidobacteriaceea* compared to the control group. The formula-fed group with the higher abundance of *Bifidobacteriaceea* required less frequent antibiotics during the first year than infants with other fecal community types. These results suggested that the anti-infectious effect of HMOs is linked to the composition of the microbiota [50].

In another trial, infants were fed from 14 days to 4 months of age with an experimental formula with a five-HMO mix (2′FL at 2.99 g/L, LNnT at 1.5 g/L, 3FL at 0.75 g/L, 6′Sl at 0.28 g/L, and 3′SL at 0.23 g/L) (*n* = 103) or a control formula (*n* = 104). In the safety outcomes, no differences were shown regarding infections and infestations [51].

Another randomized study with a similar formula (2′FL at 3 g/L, LNnT at 1.5 g/L, 3FL at 0.8 g/L, 6′SL at 0.3 g/L, and 3′SL at 0.2 g/L) showed that the experimental-formula-fed infants (*n* = 130) had less recourse to healthcare professionals for illness than the control group (*n* = 129) before 3 months of age (secondary outcomes) [52].

From 1 to 2.5 years of age (*n* = 461), the incidence of upper respiratory tract infections was similar between randomized infants receiving four different young-child formulas containing GOS (4 g/L), TGF-β (9.9 or 15 µg/L), lactoferrin (0 to 1.7 g/L), immunoglobulins (0 to 1 g/L), milk fat (0.5 to 17 g/L), and 2′FL (0 or 3 g/L). However, according to the secondary outcomes of the study, children supplemented with 2′FL had longer durations of upper respiratory tract infections and more episodes of coughs and runny noses than the group with the similar formula without 2′FL (*p* < 0.05 and *p* < 0.01, respectively). Fever episodes were less frequent, but gastrointestinal tract infections occurred more often in the group supplemented with 2′FL, immunoglobulins, and lactoferrin than in the group fed with formula without these components (*p* < 0.01 each) [53].

Whey-based extensive hydrolyzates with added HMOs (2′FL at 1 g/L and LNnT at 0.5 g/L) are free of residual milk proteins and were well-tolerated by infants allergic to cow’s milk [45]. Cow’s-milk-allergic infants in the HMO group (*n* = 94) and in the control group (*n* = 96, same formula without HMOs) had similar incidences of upper and lower respiratory tract infections, gastrointestinal infections, other viral infections, and urinary tract infections between enrollment (from 0 to 6 months) and 1 year of age. In a subanalysis, the authors evidenced a significant reduction in the frequency of upper respiratory tracts infections compared to the control group (hazard ratio: 0.58; 95% CI: [0.41–0.83]). There was a slight reduction in the occurrence of otitis media during the follow up in the HMO group. The overall uses of antibiotics and antipyretics were similar in both groups, but between the visits at 4 months for follow-up and 12 months of age, infants in the HMO group required fewer antipyretics (*p* = 0.02) [54]. There are currently no published clinical studies evidencing acceleration of the acquisition of tolerance to cow’s milk [45].

To summarize, results about the prevention of infections through HMO supplementation of infant formula are divergent, and the potential benefits of such interventions should be further studied.

#### 3.2.3. GOSs

Galacto-oligosaccharides (GOSs) are prebiotics that are more easily synthesized than HMOs, explaining why they are more frequently used in infant formulas. In vitro, they limit the adhesion of pathogens to epithelial cells and stimulate the Treg (IL10) and Th1 (increase in IFN-γ and decrease in TNF-α) pathways, inducing anti-inflammatory and regulatory effects [47]. In animals, GOSs promoted an increase in SCFAs and stimulated intestinal barrier function [47]. In infants, GOS supplementation (4.4 to 5 g/L) (*n* = 44, vs. *n* = 37 in the control group without GOS) decreased fecal pH and butyric acid concentration, whereas the effect on fecal sIgA was limited [55]. They also had bifidogenic effects [47,55,56] and reduced the gastrointestinal colonization of *Clostridium* (*n* = 83 fed with the study formula vs. *n* = 79 in the control group) [56].

Bozensky et al. studied the effect of GOS supplementation (5 g/L) in a partially hydrolyzed formula on atopic dermatitis in infants with a family history of atopy and moderate eczema at recruitment (*n* = 52 in the intervention group vs. *n* = 51 in the control group). Supplementation was provided from 6 weeks to 6 months. The SCORAD index decreased in both groups (supplemented or not), with no significant differences between the groups [57].

GOSs associated with polydextrose (PDX) (total of 4 g/L; 1:1 ratio) also had a bifidogenic effect (*n* = 91 PDX/GOS group; *n* = 91 control group; *n* = 83 breastfed group) [58] and was evidenced in increased counts of *Lactobacilli*, particularly in *L. rhamnosus*, in supplemented infants (*n* = 77), thus showing a gut microbiota closer to that of breastfed infants (*n* = 71) than to non-supplemented infants (*n* = 80) [59].

In young infants at risk of atopy, GOS/PDX supplementation (total of 4 g/L; 1:1 ratio) (*n* = 201) prevented respiratory infections in the first two years of life, with a rate similar to that observed in breastfed infants (*n* = 140) [60]. In this study, supplementation induced differences in fecal microbiota at 9–12 months of life, with increases in *Bifidobacteria* and *Clostridium* cluster I. The supplementation did not prevent atopic dermatitis, but the increased load of fecal *Bifidobacteria* at 9–12 months was associated with protection against respiratory infection. Atopic-dermatitis-free infants had higher colonization with *Clostridium* postintervention [60].

#### 3.2.4. FOSs

Fructo-oligosaccharides (FOSs) derived from inulin are also known to be bifidogenic [61,62,63], despite controversies [64]. In vitro, FOSs limit the adhesion of pathogens to intestinal cells, strengthen the intestinal barrier, and stimulate the Th1 immune pathway, as observed for GOSs [47]. Gut inflammation monitored with fecal calprotectin was not affected after 8 weeks of supplementation (3 g/L) (*n* = 10–12 infants per group; prebiotic formula, control formula, and human milk) [61] or after a 12 months of supplementation (short- and long-chain FOS and inulin combination, total of 8 g/L) (*n* = 14 fecal samples in prebiotic group and *n* = 11 in the control group) [63]. Conversely, FOSs have induced increased intestinal production of sIgA [47,63].

#### 3.2.5. GOSs/FOSs at a Ratio of 9:1

Fifteen years ago, one of the first originator studies in infants fed with a formula with GOSs/FOSs (6 g/L; GOS/FOS ratio: 9/1) (*n* = 19) showed a trend of increased rate of fecal sIgA compared to a standard formula (*n* = 19) [7]. After 1 year of intervention (4 g/L), Bruzzese et al. highlighted a reduction in digestive infections during the study period. There was a decreased number of episodes (0.12 episode per child per year vs. 0.29, *p* = 0.015), with fewer children having at least one episode of acute infectious gastroenteritis (10.4% vs. 23.9%, *p* = 0.01) and fewer children having at least two courses of antibiotics (40.0% vs. 66.2%, *p* = 0.02) (*n* = 96 in the prebiotic group; *n* = 105 in the standard formula group). Moreover, supplementation was associated with a non-significant decrease in the number of children who had at least three episodes of upper respiratory infections (28.3% vs. 44.6%, *p* = 0.06) [65].

When supplementation with GOSs/FOSs (9:1) was pursued up to 12 months of age, Shahramian et al. observed an infectious history similar to breastfed infants. The total duration of diarrhea was shorter in supplemented-formula-fed infants compared to non-supplemented (4.4 vs. 12.3 days, *p* < 0.001) and similar to that observed in breastfed infants (4.4 vs. 6.8) (*n* = 60 in each group). Additionally, GOS/FOS-supplemented infants had fewer occurrences of fever episodes and respiratory tract infections compared to regular-formula-fed infants but the same as that of breastfed infants [66].

The European Multicentric Infection Prevention Study (MIPS) demonstrated that a formula with a specific mixture of short-chain GOSs (scGOSs) plus long-chain FOSs (lcFOSs) (6.8 g/L, ratio 9:1) and pectin-derived acidic oligosaccharides (1.2 g/L) decreased the rate of atopic dermatitis by 44% in infants not considered to be at risk in their first year of life. This significant effect was not sustained at preschool age after oligosaccharide supplementation was stopped (*n* = 172 in the probiotic group) [67].

Holscher et al. studied the effect of a partially hydrolyzed whey formula supplemented with GOSs and FOSs (4 g/L, 9:1) on intestinal microbiota composition. After 6 weeks of GOS/FOS supplementation (*n* = 36), the absolute and relative quantities of *Bifidobacteria* were similar to those observed in breastfed infants (*n* = 33) and higher than those in non-supplemented infants (*n* = 33). The SCFAs (mainly acetate, propionate, and butyrate) were higher in the supplemented group than in the breastfed group. As a result, fecal pH was more acid in prebiotic and breastfed groups [68].

Another partially hydrolyzed whey protein infant formula containing scGOSs, lcFOSs (6.8 g/L; GOS/FOS ratio: 9:1), and pectin-derived acidic oligosaccharides (1.2 g/L) (*n* = 57) showed similar results in terms of bacterial taxonomic and metabolite compositions of gut microbiota close to those of breastfed infants (*n* = 30) [69]. However this formula failed to prevent eczema by 12 and 18 months in high-risk infants (*n* = 341) compared to a standard cow’s milk formula (*n* = 360) [70].

Several randomized controlled double-blind studies have focused on the use of a combination of GOSs/FOSs (8 g/L; GOS/FOS ratio = 9:1) added to an extensive whey hydrolyzate formula provided during the first 6 months of life. The aim of this formula was to prevent atopic disease in at-risk infants (at least one of the two parents having atopy). At 6 months, the cumulative incidence of atopic dermatitis was lower in the supplemented group (9.8% vs. 23.1%, *p* = 0.014, total *n* = 206) [71]. In a subgroup of 84 children, the supplemented infants had significantly lower totals of IgE, IgG1, IgG2, and IgG3 antibody concentrations in serum than non-supplemented infants [72]. A gut microbiota analysis revealed an increase in the number of *Bifidobacteria* at 6 months under GOS/FOS supplementation (subgroup of 98 children) [71]. At 2 years of age (*n* = 134), a significant reduction in the cumulative incidence of allergic manifestations was observed (atopic dermatitis: 13.6% vs. 27.9%; recurrent wheezing: 7.6% vs. 20.6%; urticaria: 1.5% vs. 10.3%) [73]. Supplemented infants also had fewer episodes of upper respiratory infections and fevers and fewer courses of antibiotics [73]. At 5 years (*n* = 92), i.e., 4.5 years after stopping the prebiotics, a lower cumulative incidence of allergic manifestations was still observed in the supplemented group (30.9% vs. 66.0%, *p* < 0.01), with notably less atopic dermatitis [74].

#### 3.2.6. GOSs and/or FOSs

After 4 months of supplementation with GOSs (0.6 g/100 g), FOSs (0.8 g/100 g), and 1,3-olein-2-palmitin (OPO) (4 g/100 g), the most abundant triacylglycerol in breastmilk, (*n* = 22 in the supplemented formula group), the alpha diversity and richness of gut microbiota decreased compared to infants fed with regular formula (*n* = 13), approximating that of breastfed children (*n* = 48) [75]. GOS/FOS/OPO supplementation was associated with a beta diversity (meaning the phylogenetic distance between samples) closer to that of breastfed infants, with a higher relative abundance of *Enhydrobacter* and *Akkermansia* [75]. In terms of microbiota metabolism functions, supplemented children and breastfed children had similar proportions of intestinal bacteria related to septicemia and ureolysis [75].

In an ELFE cohort, no association was observed between the consumption of GOSs/FOSs or GOSs only at 2 months and the occurrence of respiratory disease up to 5.5 years. Nevertheless, early use of GOSs was associated with a lower risk of upper respiratory tract infections compared to infants never supplemented with GOSs (OR: 0.87; 95% CI: [0.76–0.99]) [10].

The main clinical effects of prebiotics in infant formula are summarized in Table 2.

### 3.3. Synbiotics

#### 3.3.1. Definition

A synbiotic is a “mixture comprising live microorganisms and substrate(s) selectively utilized by host microorganisms that confers a health benefit on the host” [76]. The synergistic and the complementary effects of the substrate, which is not necessarily a prebiotic, make it possible to gain the effects of the probiotic and the substrate as a costimulant on the microbiota and immune functions. Yogurt is the archetype synbiotic food in lactose intolerance, with a health claim recognized by the EFSA [77].

#### 3.3.2. *Bifidobacterium animalis* sp. *lactis* Bb-12 and Bovine-Milk-Derived Oligosaccharides (BMOs)

BMOs are natural prebiotics derived from cow’s milk, and some have identical or similar structures to HMOs. The most-used BMOs are GOSs and 3′- and 6′-sialyllactose.

Over a 12-week period, supplementation of a standard cow’s milk formula with *B. lactis* (10^7^ CFU/g of powder formula) and BMOs (8 g/L of reconstituted formula) in healthy infants (*n* = 37 in the test formula group; *n* = 37 in the control group without supplementation) induced a fecal composition close to that observed in breastfed children (*n* = 39), particularly with a similar amount of *Lactobacillus* and an intermediate level of *Bifidobacterium*. Alpha diversity was lower in both the intervention group and breastfed infants than in the control group (significant difference up to 6 weeks, then at an intermediate level for the intervention group at 12 weeks). Moreover, the supplementation induced a microbiota shift toward a *Bifidobacterium*-dominated fecal microbiota [78]. After 3 and 6 months of supplementation, *Bifidobacteria* and *Lactobacillus* counts were higher in the stool of supplemented infants than in control group or breastfed infants. Stool pH and sIgA were intermediate in the intervention group [79]. However, at 6 and 12 months of life, the proportions of infants who experienced at least one episode of diarrhea or other febrile infection were similar among the three groups (test formula, *n* = 179; control formula, *n* = 180; breastfed, *n* = 59) [79].

A European study randomized 127 healthy infants in a controlled clinical trial. During the first 8 weeks of life, infants received either a standard formula (*n* = 40), a formula supplemented with native bovine lactoferrin (1 g/L) plus probiotics (*B. lactis* Bb-12 3.7 ± 2.1 × 10^4^ CFU/g of powder formula) (FLP) (*n* = 39), or the same supplemented formula plus prebiotics (3’ and 6’-sialyllactose oligosaccharides at a concentration of 6 g/L) (FLPP) (*n* = 35). Children fed with FLPP had the lowest fecal calprotectin concentrations compared to breastfed children (*n* = 61) (*p* = 0.012 at 8 weeks), meaning lower gut inflammation. During the intervention period, the *Bifidobacterium* genus predominated in the stool of children on FLPP (77%), as observed in breastfed infants (81%) but not in the other groups (part of the cohort for the microbiota analysis). However, the predominance of this bacterial genus quickly disappeared after the discontinuation of these supplemented formulas [80].

#### 3.3.3. *B. lactis animalis* sp. *lactis* Bb-12 and GOSs/FOSs

No differences were noticed regarding infections (upper and lower respiratory tract and gastrointestinal infections) and antibiotic use during the first year of life in infants receiving, from the first month of life to 12 months, formulas containing *B. lactis* Bb-12 (10^7^ CFU/g) with or without GOSs/FOSs (4 g/L; GOS/FOS ratio: 9/1) (*n* = 219 in the Bb-12 group; *n* = 220 in the Bb-12 with GOSs/FOSs group) [81].

#### 3.3.4. *Bifidobacterium breve* (Bb) M-16V and GOSs/FOSs

The *Bb* M-16V strain alone had digestive anti-inflammatory properties, restored intestinal tight junctions, conferred protection against ulcerative-necrotizing enterocolitis in premature babies, and had anti-allergic properties (asthma, food allergies, and respiratory allergies), as demonstrated in vitro and in vivo in animals (decrease in specific IgE, modulation of the protolerogenic Th1–pro-allergic Th2 balance) [82]. To our knowledge, Bb M-16V is not used alone as a probiotic in infant formula, but mostly used as an addition into diets through milk or water [82].

In healthy infants born by caesarean section and receiving complementary feeding with a standard infant formula, supplementation with *Bifidobacterium breve* M-16V (7.5 × 10^8^ CFU/100 mL) and GOSs/FOSs (8 g/L) (*n* = 52) led to a faster fecal implantation of *Bifidobacteria* compared to similar infants not receiving a synbiotic (*n*= 50) or receiving prebiotics only (*n* = 51). In synbiotic-supplemented infants, this parameter was comparable to that observed in infants delivered vaginally (*n* = 30) [83]. In the first days of the intervention, the synbiotic formula modulated the anaerobic catabolism in the guts of infants delivered by C-section compared to infants delivered vaginally [84]. In a post hoc analysis, infants fed with complementary synbiotics had less atopic dermatitis than infants fed with standard formula [83]. Beyond the age of 1 year, a growing-up infant formula supplemented with a synbiotic based on Bb M-16V (1.8 × 10^7^ CFU/g) and GOSs/FOSs (9.5 g/L) also showed an increase in the proportion of *Bifidobacterium* in the fecal microbiota associated with stool acidification [85].

The effects of supplementation with an extensive whey hydrolyzate with Bb M-16V (1.3 × 10^9^ CFU/100 mL) associated with a GOS/FOS mixture (8 g/L, 9:1) were evaluated in infants younger than 7 months with moderate-to-severe atopic dermatitis (SCORAD > 15) [86,87]. After 12 weeks of intervention, the children in the synbiotic group (*n* = 46) had a higher relative proportion of *Bifidobacteria* (54.7% vs. 30.1%, *p* < 0.001) and fewer bacteria with pathogenic potential (*Clostridium lituseburense-C. histolyticum* and *Eubacterium rectale-C. coccoides*) compared to children who were fed with the non-supplemented extensive hydrolyzate (*n* = 44) [86]. The fecal metabolic profile of supplemented infants was different from that of the placebo group (lower pH, higher lactate concentrations, and lower butyrate, isobutyrate, and isovalerate concentrations) [86]. At 12 weeks and at 1 year, the authors did not observe any effect on the severity of atopic dermatitis, with an improvement in the SCORAD indices of the two groups [86,87]. However, galectin-9, a protein expressed by intestinal epithelial cells, increased in serum. This protein may play a role in reducing the severity of allergies and in acquiring allergen tolerance, but this was only evidenced in mice [88]. However, no effect of the synbiotic supplementation was observed for other soluble biomarkers (IL-5, IgG1, IgG4, CTACK, and TARC), ex vivo cytokine production by stimulated PBMCs, or Treg percentage [89].

At the 1-year follow up, the supplemented children had fewer reports from parents of asthmatic symptoms (more than three wheezing episodes over the period and wheezing or noisy breathing apart from colds) and had less recourse to anti-asthmatic treatments (significant reduction in absolute risk between 19.4 and 28.0 depending on the studied parameter) [87].

A series of randomized studies have investigated the effects of an amino-acid-based formula supplemented with Bb M-16V (1.47 × 10^9^ CFU/100 mL) and prebiotics (oligo-fructose and long-chain inulin, total of 6.3 g/L, ratio of 9:1) (AAF-Syn) given for a period from 8 weeks to 12 months in infants with IgE- and non-IgE-mediated cow’s milk protein allergies (PRESTO studies) [90,91,92,93,94]. Compared to infants fed with amino acid formula without synbiotics, children on AAF-Syn had more *Bifidobacterium spp.* and *Veillonella spp.*, lower bacterial diversity, and correction of the *Eubacterium rectale/Clostridium coccoides* ratio in stool similar to digestive microbiota of breastfed infants, with an improvement in dysbiosis [90,94]. According to a meta-analysis published in 2021, these infants (pooled results, AAF-Syn: *n* = 169; AAF alone, *n* = 180) also had significantly fewer infections (overall reduction of 51%), less use of antibiotics, and fewer hospitalizations (56% reduction) [90]. In the latest publication about the PRESTO studies, fewer infants were hospitalized for serious adverse events due to infections (9% in AAF-Syn, *n* = 7/80 vs. 20% in AAF, *n* = 18/89; *p* = 0.036) [94]. According to primary outcomes, the age of acquisition of tolerance toward cow’s milk protein was similar with or without the synbiotics [94].

#### 3.3.5. *Lactobacillus fermentum* CECT5716 and GOSs

According to the secondary outcomes of a randomized controlled trial comparing supplementation from 1 to 6 months of life with *L. fermentum* CECT5716 (10^7^ CFU/g) plus GOSs (3 g/L) (*n* = 61) vs. GOSs only (3 g/L) (*n* = 60), infants in the synbiotic group had a significant reduction in the incidence rate of gastrointestinal infections and diarrhea (−71%, incidence rate ratio: 0.289, *p* = 0.018) [95]. In a follow-up study, protection from infections was not maintained at 3 years of age (*n* = 45 in the synbiotic group; *n* = 55 in the prebiotic group) [96].

When a synbiotic follow-on formula (*L. fermentum* CECT5716 (average of 2 × 10^8^ CFU/day) and GOSs at 4 g/L) was given to infants from 6 to 12 months old, bacterial counts of *Lactobacilli* and *Bifidobacteria* were significantly higher in the synbiotic group (*n* = 97) than in the prebiotic group (*n* = 91) at 12 months. No significant differences were seen in fecal SCFAs and sIgA concentrations in fecal samples. From a clinical point of view, the main outcomes of this study revealed that infants in the synbiotic group had 46% fewer gastrointestinal infections than the control group (incidence rate ratio: 0.54, *p* = 0.032) and 26% fewer respiratory infections (mainly upper respiratory infections) than the control group (incidence rate ratio: 0.74, *p* = 0.022) [97].

Whenever a synbiotic formula was used (from 1 to 6 months or from 6 to 12 months), no significant differences were found for other infections, antibiotic use, or fever episodes [95,97].

#### 3.3.6. *Lactobacillus reuteri* DSM 17,938 and 2′FL

The synbiotic combination of *L. reuteri* (10^7^ CFU/g) and 2′FL (1 g/L) up to 6 months of age led to an increased fecal *Bifidobacterium* proportion in the interventional group (*n* = 144) compared to the control group (*L. reuteri* without 2′FL) (*n* = 145) similar to the fecal microbiota of breastfed infants, and reflecting the bifidogenic effect of 2′FL. At 1 month of age, opportunistic pathogens, such as *Clostridioides difficile*, were significantly less abundant in feces from infants receiving synbiotics compared to probiotics but similar to feces from breastfed infants (*n* = 60) [46]. Microbiota alpha diversity was significantly higher in the synbiotic group than in breastfed infants [46], but beta diversity suggested a shift in the microbiota composition of the synbiotic group toward that of breastfed infants. Acetate and propionate concentrations were higher and lactate was lower in both formula groups compared to the breastfed group [46].

#### 3.3.7. *Bifidobacterium longum* ATCC BAA-999 (Bl999) and *Lactobacillus rhamnosus* CGMCC 1.3724 (LPR) ± BMOs

After 2 months of supplementation (Bl999 and LPR, 2 × 10^7^ CFU/g each, and BMOs at 10 g/L or none (in the case of the control group)) from birth, *Bifidobacteria* was detectable in most infant fecal samples, whatever the intervention group: BMOs with probiotics formula (*n* = 98) (100%), BMO formula (*n* = 99) (83.3%), and non-supplemented formula (*n* = 84) (79.2%). *Bifidobacteria* had, nevertheless, a higher significant count in the first group. *Lactobacillus* species were more frequently detected and more abundant in stool from the BMO and probiotics group compared to those from the BMO group (16.7%) and the control group (8.3%), meaning that probiotics had a greater lactobacillogenic effect than the prebiotic BMOs only. *Clostridia* were less abundant in the BMO and probiotics + BMO groups than in the control group (*p* < 0.05) [98].

#### 3.3.8. *Lactobacillus paracasei* sp. *paracasei* strain F19 and GOSs/FOSs

A comparison between a prebiotic formula (GOSs at 5.4 g/L; FOSs at 0.61 g/L) (*n* = 92) and a synbiotic formula (F19 10^9^ CFU/L and GOSs/FOSs) (*n* = 90) in full-term infants from day 28 to month 4 of life showed a significant reduction in the relative risk of lower respiratory tract infections during the 0–12-month period in favor of the synbiotic formula (RR: 0.34; 95%CI: 0.13–0.85). However, this point was a secondary outcome, and the study was not sufficiently resourced [99].

#### 3.3.9. *Lactobacillus rhamnosus* LCS- 742, *Bifidobacterium longum* sp. *infantis* M63, and GOSs/FOSs

Non-breastfed full-term newborns were randomized between a synbiotic formula (*L. rhamnosus* LCS-742 at 1.4 × 10^8^ CFU/100 mL, *B. longum sp. infantis* M63 at 1.4 × 10^8^ CFU/100 mL, GOSs at 4 g/L, and FOSs at 0.2 g/L) (*n* = 48) and a non-supplemented formula (*n* = 49). After 6 months, the experimental formula prevented the occurrence of atopic dermatitis (1/39 vs. 8/45, *p* = 0.03). Fecal sIgA concentrations were maintained over time in the synbiotic group compared to those of controls, which decreased. The more significant this decrease, the greater the risk of developing atopic dermatitis. The decline in sIgA was negatively correlated to the colonization of *Bifidobacteria* [100].

#### 3.3.10. *B. infantis* IM1, *L. rhamnosus* LCS-742, FOSs, and Inulin

Gut microbiota maturation was explored in healthy infants from 0–2 months to 18 months of age, either breastfed (*n* = 42) or randomized to receive a synbiotic formula (*B. infantis* IM1: 10^7^ CFU/g; *L. rhamnosus* LCS-742: 10^7^ CFU/g; FOSs and inulin: total of 2.6 to 2.7 g/L, ratio of 1:1; bovine milk fat globule membranes (MFGMs), and long-chain polyunsaturated fatty acids (LC-PUFAs)) (*n* = 69) or a standard formula (no synbiotics, no MFGMs, no LC-PUFAs) (*n* = 60). Cerdó et al. evidenced several microbial enterotypes associated with age and type of feeding, as well as with mode of delivery, daycare, and pre-pregnancy maternal body mass index. Before 12 months of age, species richness was significantly higher in formula-fed infants than in the breastfed group. The synbiotic formula was associated with a higher abundance in *Lactobacillus* compared to the standard formula group. Regarding the *Bifidobacterium* genus, although the abundance was similar between the two infant formulas, time- and species-specific effects were observed [101].

Table 3 sums up the observed clinical outcomes of synbiotics in infant formula.

### 3.4. Postbiotics

#### 3.4.1. Definition

Postbiotics are a “preparation of inanimate microorganisms and/or their components that confers a health benefit on the host”. They are “deliberately inactivated microbial cells with or without metabolites or cell components that contribute to demonstrated health benefits” [102].

#### 3.4.2. Postbiotics Produced by *Lactobacillus paracasei* (CBA L74)

Several Italian teams have taken an interest in postbiotics resulting from the fermentation of skimmed milk with *Lactobacillus paracasei* (CBA L74), a strain isolated from the feces of healthy infants [103,104,105,106]. In brief, the fermented milk was prepared from skimmed milk fermented with 10^6^ CFU of *L. paracasei* CBA L74/g. The bacterial growth was stopped after 15 h of incubation at 37 °C when the bacteria reached 5.9 × 10^9^ CFU/g, and the bacteria was inactivated with a quick heating. An initial study in mice showed a protective effect of milk fermented using *Lactobacillus paracasei* (CBA L74) in induced colitis, protection against pathogens (*Salmonella*), and inhibition of pro-inflammatory cytokines in favor of anti-inflammatory cytokines [104]. The active components were the metabolites from the fermentation and not the live or killed bacteria [104]. Then, thanks to a skim cow’s milk fermented with *L. paracasei* L74 (not infant formula), the authors reported fewer common infections (in particular, acute gastroenteritis, pharyngitis, laryngitis, and tracheitis) and less use of drugs (antipyretics, antibiotics, and corticosteroids) in children from 12 to 48 months of age supplemented over a period of 3 months. Immuno-stimulation has been demonstrated, with increases in concentrations of fecal peptides and proteins (α-defensin, β-defensin, sIgA, and cathelicidin LL-37) resulting from the activation of the innate and acquired immune system [105,106]. Finally, this principle of fermentation (fermented spray-dried milk for infant milk tins) was applied to an infant formula administered to newborns up to 3 months of age (three groups: intervention, control, and breastfed; *n* = 26 in each group) [103]. Infants receiving the fermented formula had a similar microbiota to that of breastfed infants, namely a reduction in fecal bacterial diversity, an intermediate level of sIgA, and a metabolomic profile close to that of breastfed infants. However, over the period studied, unlike the studies by Corsello et al. [105] and Nocerino et al. [106], no difference in antimicrobial peptides was observed [103].

#### 3.4.3. Postbiotics Produced by *Bifidobacterium breve* C50 and *Streptococcus thermophilus* ST065

*Bifidobacterium breve* C50 and *Streptococcus thermophilus* ST065 are lactic-acid-producing bacteria with anti-inflammatory properties on intestinal cells in vitro [107]. A fermented infant formula based on these two strains with no living bacteria in the final product was tested in healthy infants (*n* = 464) and compared to infants receiving non-supplemented formula (*n* = 449). Fermented and control formula were provided for 5 months after the age of 4 months. While the incidence of acute diarrhea was the same in both groups, the severity of acute gastroenteritis was less in the fermented milk group, with fewer hospitalizations, fewer cases of acute dehydration, fewer medical consultations, and fewer prescriptions for oral rehydration solutions [108]. Between 6 and 24 months of age, the incidence of cow’s milk protein allergy was the same in both groups (*n* = 66 and 63 in the fermented milk group and standard formula group, respectively), but sensitization to milk assessed by skin prick tests and digestive or respiratory symptoms of suspected allergy were lower in infants at high risk of atopy receiving the postbiotic-supplemented formula [109]. The fecal pH was similar from day 3 of life to 4 months in newborns for infants fed the fermented milk (*n* = 30) and breastfed (*n* = 30) and was more acidic than in infants fed the standard formula (*n* = 30) [110].

When this fermented formula (containing 0.25 g of 3′-galactosyllactose/L) was combined with GOSs/FOSs (8 g/L, ratio of 9:1) (*n* = 30), fecal sIgA concentrations and the compositions of the fecal microbiota were similar to those of breastfed infants (*n* = 30) [111,112]. Nevertheless, untargeted metabolomic profiles remained distinct, even if stable over time, between infants fed with pre- and postbiotic-supplemented formula and breastfed infants, with 261 different metabolites at the end of the study (vs. 404 different metabolites between the control formula and the breastfed group) [112].

#### 3.4.4. Postbiotics produced by *Bifidobacterium animalis* sp. *lactis* CECT 8145 BPL1^TM^

According to secondary outcomes of the INNOVA 2020 study, infants randomized to be fed with an intervention formula (containing a thermally inactivated postbiotic, BPL1^TM^, and a lower amount of protein, a lower casein-to-whey protein ratio, and a double amount of docosahexaenoic acid/arachidonic acid compared to a standard formula) (*n* = 70) exhibited less atopic dermatitis and fewer bronchitis and bronchiolitis episodes than infants in the standard group (*n* = 70) (*p* = 0.03). These rates were similar as in breastfed children (*n* = 70) (*p* = 1.0). Other morbidities, such as infections, were not different among the three groups during the timeframe of the study [113].

The clinical effects of infant formula with postbiotics are summarized in Table 4.

## 4. Discussion and Conclusions

**Figure 2:**
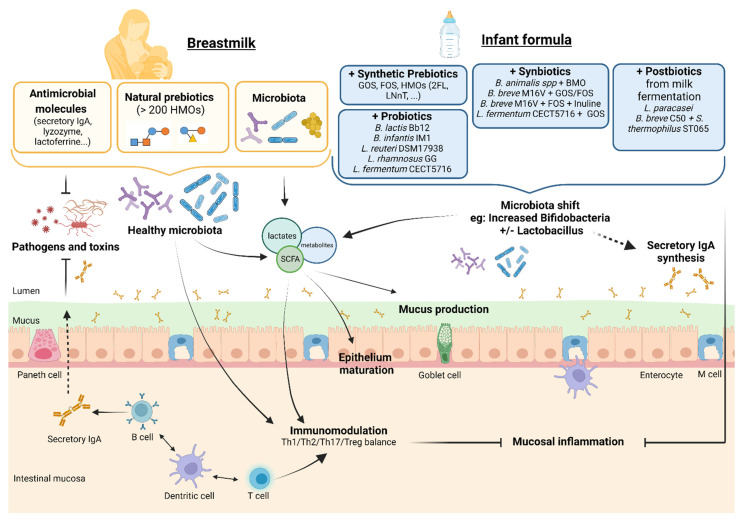
Supposed effects on the intestinal barrier, immunity, and microbiota of infant formula supplemented with pre-, pro-, syn-, and postbiotics compared to breastfeeding. Legend: BMOs—bovine-milk-derived oligosaccharides; FOSs—fructo-oligosaccharides; GOSs—galacto-oligosaccharides; HMOs—human milk oligosaccharides.

There have been many advances in recent years to try to improve the composition of infant formula so that the microbiota and immunity of non-breastfed infants are as close as possible to those of breastfed infants. Figure 2 sums up the main effects of the “-biotic” formulas.

Some probiotics (*Bb12*, *B. animalis* sp. *lactis* HN019, *L. fermentum* CECT5716, and LGG) have demonstrated positive health effects in infants, notably in preventing infancy infections [10,15,34,36], in improving atopic dermatitis, or in accelerating food allergy remission [22,23,24,25,26]. Some bacteria can modulate the whole microbiota composition and digestive microenvironment (pH, metabolites, etc.). These probiotics only represent fractions of the microbiota and cannot restore a “non-dysbiotic” microbiota by themselves, as with the theorical microbiota of infants delivered vaginally and breastfed [4].

Clinical beneficial effects of prebiotics (mainly GOSs, FOSs, and HMOs) have been observed, particularly, to effect a modest reduction in infections in infants [7,49,52,54,60,66], with no relevant effect in others [43,51,53,55,57,63,114]. Prebiotics may prevent the occurrence and severity of atopic dermatitis [67,71,115].

Regarding synbiotics (*Lactobacillus fermentum* CECT 5716 and GOSs; *B. breve* M16V and GOSs/FOSs; *Lactobacillus rhamnosus* LCS- 742, *Bifidobacterium longum* sp. *infantis* M63, and GOSs/FOSs) and postbiotics (*L paracasei* CBA L74; *Bifidobacterium breve* C50 and *Streptococcus thermophilus* ST065; *B. animalis* sp. *lactis* CECT 8145 BPL1^TM^), some results appear interesting in terms of preventing infancy infections and atopic diseases [87,90,94,95,97,99,100,105,106,108,109,113]. As for pre- and probiotics, the main proven effects are shifts in microbiota composition to be closer to that of breastfed infants.

Unfortunately, it seems difficult to provide a meta-analysis and then strong recommendations with a high quality of evidence for the potential immune and clinical effects of pre-, pro-, syn-, and postbiotics in infant formula. Each formula does indeed have different nutritional components, different sources and molecular weights of cow’s milk proteins, different prebiotic or probiotic strains, and several doses, as well as different targets in terms of infants and their period of life. All these variable parameters may explain the apparent divergent results among studies. Despite the effects demonstrated in vitro or in animals and the rich literature of randomized controlled studies, the chosen primary or secondary outcomes in trials are not always relevant for clinical practice. Clinical trials should mainly focus on the mid- to long-term effects on the microbiota rather than the short-term effects. Moreover, although impacts on the bacterial microbiota are increasingly observed, the mechanisms and the long-term positive or negative consequences on microbiota function, immunity, and the metabolomic profiles of these pre-, pro-, syn-, and postbiotics given early in life when the microbiota and the immune system are still immature need to be clarified.

There is currently not enough robust evidence to recommend the routine use of these “-biotics” in infant formulas in healthy or atopic infants who cannot be breastfed [13,43,116,117]. To date, there is no perfect formula that combines all the “ingredients” to exactly mimic and reproduce all the benefits of breastmilk, which itself varies interindividually (from one mother to another) and over time according to the ages of newborns and infants. The choice of infant formula is a decision made by parents from a very wide range on the market, possibly after informed advice from their doctor, pediatrician, or pharmacist, based on rather strong evidence of efficacy in line with the scientific literature.

## Figures and Tables

**Table 1:** Summary of clinical effects of probiotics compared to control groups with non-supplemented infant formula.

Probiotics	Dose and Duration	Clinical Effects	References
Bb-12	10^6^ CFU/g in fermented and acidified formula (*S. thermophilus and L. helveticus*) T0: before 8 months of life Period: at least 4 months	Lower incidence of acute gastroenteritis	[15]
	10^4^ to 10^7^ CFU/g Period: 0–12 months of age	Similar prevalence of acute gastroenteritis before 6 months of age	[17]
Bb-12 with *L. casei CRL431*	10^7^ CFU/g each (in extensively hydrolyzed casein formula) T0: before 6 months Duration: 6 months	Similar duration of cow’s milk allergy	[18]
*L. reuteri* DSM 17938	retrospective observational cohort	No prevention of respiratory diseases up to 5 years of age	[10]
LGG	1.4 × 10^7^ CFU/100 mL (in extensively hydrolyzed casein formula) Start: 1–12 months of age Duration: until acquisition of tolerance to cow’s milk	- Accelerated acquisition of tolerance to cow’s milk - Improved functional bowel disorders in cow’s-milk-allergic patients - Limited atopic march before 3 years of age - No prevention of infections	[22,23,24,25,26]
*L. fermentum* CECT5716	10^7^ CFU/g Period: 1–12 months of age	Lower incidence and shorter duration of diarrhea	[34]
	retrospective observational cohort	Higher risk of upper respiratory tract infection if consumption discontinued between 2 and 10 months of age, whereas no additional risk for daily consumption	[10]
*B. breve* CECT7263	10^7^ CFU/g Period: 1–12 months of age	Similar incidence and duration of respiratory and gastrointestinal infections during the first year of life	[34]
*B. infantis* IM1	10^7^ CFU/g Start: before 3 months of life Duration: 12 weeks	No significant effect on diarrhea	[35]
*B. animalis* sp. *lactis* HN019	10^6^ CFU/g Start: 6–12 months of age Duration: 12 weeks	Fewer physician-confirmed infections and fewer parentally reported infections	[36]
*L. rhamnosus HN001*	10^6^ CFU/g Start: 6–12 months of age Duration: 12 weeks	No significant effect on infections	[36]
Mix of *Bifidobacteria* (*B. bifidum*, *B. breve*, *B. longum*, and *B. longum* sp. *infantis*)	10^7^ CFU/g Period: 0–12 months of age	No significant effect on episodes of fever, diarrhea, or antibiotics recourse	[37]

**Table 2:** Summary of clinical effects of prebiotics compared to control groups with non-supplemented infant formula.

Prebiotics	Dose and Duration	Clinical Effects	References
HMOs	2′FL (1 g/L) and LNnT (0.5 g/L) Period: 0–6 months of age	Fewer respiratory infections, less use of antibiotics and antipyretics before the age of 1 year	[49]
	5-HMO mix (2′FL at 2.99 g/L, LNnT at 1.5 g/L, 3FL at 0.75 g/L, 6′SL at 0.28 g/L, and 3′SL at 0.23 g/L) Period: 0–4 months of age	No significant effect on infections and infestations	[51]
	2′FL at 3 g/L, LNnT at 1.5 g/L, 3FL at 0.8 g/L, 6′SL at 0.3 g/L, and 3′SL at 0.2 g/L Period: 0–4 months of age	Less recourse to healthcare professionals for illness before 3 months of age	[52]
	Combination of GOSs (4 g/L), TGF-β (9.9 or 15 µg/L), lactoferrin (0 to 1.7 g/L), immunoglobulins (0 to 1 g/L), milk fat (0.5 to 17 g/L), and 2′FL (0 or 3 g/L) (4 groups) Period: 1–2.5 years of age	- Similar rates of upper respiratory tract infections among the 4 groups - Longer duration of upper respiratory tract infections and more episodes of coughs and runny noses (if 2′FL) - Fewer fever episodes (if 2′FL with immunoglobulins and lactoferrin) - Fewer gastrointestinal tract infections (if 2′FL with immunoglobulins and lactoferrin)	[53]
	2′FL at 1 g/L and LNnT at 0.5 g/L (in extensive whey protein hydrolyzate) Start: 0–6 months of age End: 12 months of age	- Similar incidences of upper and lower respiratory tract infections, gastrointestinal infections, other viral infections, and urinary tract infections before 1 year of age - Fewer upper respiratory tract infections (subanalysis) - Slight reduction in occurrence of otitis media - Similar overall use of antibiotics and antipyretics - Less antipyretic use between 4-month follow up and 12 months of age	[54]
GOSs	5 g/L (in partially hydrolyzed formula) Period: 1–6 months of age	No specific effect of GOSs on atopic dermatitis	[57]
	GOSs at 2 g/L with PDX at 2 g/L Period: 0–11 months of age	- Similar rate of respiratory infections in infants at risk of atopy in the first two years of life as that of breastfed children - No prevention of atopic dermatitis	[60]
	retrospective observational cohort	Lower risk of upper respiratory tract infections up to 5.5 years of age with early consumption of GOSs compared to infants never supplemented	[10]
GOS/FOS Ratio of 9:1	4 g/L Start: 0–4 months of age End: 12 months of age	- Fewer digestive infections - Fewer children having ≥ 2 courses of antibiotics	[65]
	? g/L Period: 0–12 months	- Shorter duration of diarrhea - Fewer fever episodes and respiratory tract infections	[66]
	6.8 g/L Start: before 2 months of age End: 12 months of age	Decreased rate of atopic dermatitis in the first year of life; no sustained effect after stopping supplementation	[67]
	6.8 g/L with acidic oligosaccharide at 1.2 g/L (in partial whey protein hydrolyzate) Period: 0–6 months of age	No prevention of atopic dermatitis at 12 months	[70]
	8 g/L Period: 0–6 months of age	- Fewer episodes of upper respiratory infections, fevers, and courses of antibiotics before 2 years of age - Lower incidence of allergic manifestations, included atopic dermatitis, before 5 years of age	[73,74]
GOSs/FOSs	retrospective observational cohort	No association between consumption of GOSs/FOSs at 2 months and occurrence of respiratory disease up to 5.5 years of age	[10]

**Table 3:** Summary of clinical effects of synbiotics compared to control groups with non-supplemented infant formula.

Synbiotics	Dose and Duration	Clinical Effects	References
Bb-12 with oligosaccharides	10^7^ CFU/g with BMOs at 8 g/L Period: 0–6 months of age	Similar rates of diarrhea and febrile infections	[79]
	10^7^ CFU/g with GOSs/FOSs at 4g/L (ratio of 9:1) Period: 0–12 months of age	Similar rates of respiratory tract and gastrointestinal infections and antibiotic use	[81]
Bb M-16V with oligosaccharides	7.5 × 10^8^ CFU/100 mL with GOSs/FOSs (8 g/L) Period: 0–3 months of age	Less atopic dermatitis	[83]
	1.3 × 10^9^ CFU/100 mL with GOSs/FOSs (8 g/L, 9:1) Start: before 7 months of age Duration: 12 weeks	- Reduced severity of atopic dermatitis in both groups - Fewer asthmatic symptoms and less recourse to anti-asthmatic treatment until 1 year of age	[86,87]
	1.47 × 10^9^ CFU/100 mL with FOSs and long-chain inulin (6.3 g/L, 9:1) (in amino-acid-based formula) Period: 0–12 months of age Duration: from 8 weeks to 12 months	- Less use of antibiotics - Fewer hospitalizations (included for serious adverse infectious events) - Similar age of resolution of cow’s milk protein allergy	[90,91,92,94]
*L. fermentum* CECT5716 with GOSs	10^7^ CFU/g with 3 g/L Period: 1–6 months of age	- Fewer gastrointestinal infections and diarrhoea episodes before 6 months (effect not maintained at 3 years of age) - No significant effects on other infections, antibiotic use, or fever episodes	[96]
	2 × 10^8^ CFU/day with 4 g/L Period: 6–12 months of age	- Fewer gastrointestinal and respiratory infections - No significant effects on other infections, antibiotic use, or fever episodes	[97]
*L. paracasei* F19 with GOSs/FOSs	10^9^ CFU/L with GOSs at 5.4 g/L and FOSs at 0.61 g/L Period: 1–4 months of age	Fewer respiratory tract infections during the 0–12-month period	[99]
*L. rhamnosus LCS-742* with *B. longum* sp. *infantis* M63 and GOSs/FOSs	LCS-742 at 1.4 × 10^8^ CFU/100 mL, M63 at 1.4 × 10^8^ CFU/100 mL, GOSs at 4 g/L, and FOSs at 0.2 g/L Period: 0–6 months	Less occurrence of atopic dermatitis	[100]

**Table 4:** Summary of clinical effects of postbiotics compared to control groups with non-supplemented infant formula.

Postbiotics	Duration	Clinical Effects	Reference
*B. breve C50* with *S. thermophilus ST065*	Start: 4–6 months of age Duration: 5 months	Lower severity (hospitalization, dehydration, medical consultations, prescription for oral rehydration solutions) but similar incidence of acute gastroenteritis	[108]
	Period: 0–12 months of age	Less cow’s milk sensitization and fewer digestive or respiratory allergic symptoms	[109]
*B. animalis* sp. *lactis* CECT 8145 BPL1^TM^	Period: 0–12 months of age	Less atopic dermatitis and fewer bronchitis and bronchiolitis episodes	[113]

## Data Availability

Not applicable.
